# Agreement of 2 electrolyte analyzers for identifying electrolyte and acid‐base disorders in sick horses

**DOI:** 10.1111/jvim.15889

**Published:** 2020-09-23

**Authors:** Diego E. Gomez, Sébastien Buczinski, Shannon Darby, Megan Palmisano, Sarah S. K. Beatty, Robert J. Mackay

**Affiliations:** ^1^ Department of Clinical Studies, Ontario Veterinary College University of Guelph Guelph Ontario Canada; ^2^ Department of Clinical Studies, College of Veterinary Medicine University of Florida Gainesville Florida USA; ^3^ Department of Comparative, Diagnostic & Population Medicine, College of Veterinary Medicine University of Florida Gainesville Florida USA; ^4^ Département de Sciences Cliniques, Faculté de Médecine Vétérinaire Université de Montréal Montréal Quebec Canada

**Keywords:** equine, physicochemical approach, strong ion difference, unmeasured anions

## Abstract

**Background:**

Use of different analyzers to measure electrolytes in the same horse can lead to different interpretation of acid‐base balance when using the simplified strong ion difference (*s*SID) approach.

**Objective:**

Investigate the level of agreement between 2 analyzers in determining electrolytes concentrations, *s*SID variables, and acid‐base disorders in sick horses.

**Animals:**

One hundred twenty‐four hospitalized horses.

**Methods:**

Retrospective study using paired samples. Electrolytes were measured using a Beckman Coulter AU480 Chemistry analyzer (PBMA) and a Nova Biomedical Stat Profile (WBGA), respectively. Calculated *s*SID variables included strong ion difference, SID_4_; unmeasured strong ions, USI; and total nonvolatile buffer ion concentration in plasma (A_tot_). Agreement between analyzers was explored using Passing‐Bablok regression and Bland‐Altman analysis. Kappa (κ) test evaluated the level of agreement between analyzers in detecting acid‐base disorders.

**Results:**

Methodologic differences were identified in measured Na^+^ and Cl^−^ and calculated values of SID_4_ and USI. Mean bias (95% limits of agreement) for Na^+^, Cl^−^, SID_4_, and USI were: −1.2 mmol/L (−9.2 to 6.8), 4.4 mmol/L (−4.4 to 13), −5.4 mmol/L (−13 to 2), and −6.2 mmol/L (−14 to 1.7), respectively. The intraclass correlation coefficient for SID_4_ and USI was .55 (95%CI: −0.2 to 0.8) and .2 (95%CI: −0.15 to 0.48), respectively. There was a poor agreement between analyzers for detection of SID_4_ (κ = 0.20, 95%CI, 0.1 to 0.31) or USI abnormalities (κ = −0.04, 95%CI, −0.11 to 0.02).

**Conclusions and Clinical Importance:**

Differences between analyzer methodology in measuring electrolytes led to a poor agreement between the diagnosis of acid‐base disorders in sick horses when using the *s*SID approach.

**Abbreviations**
A^−^: total net negative charge of plasma proteins
AG: anion gap
A_tot_: total plasma concentration of nonvolatile weak acids
HCO_3_^−^: bicarbonate
ICC: intraclass correlation coefficient
L‐lac^−^: l‐lactate
P_v_CO_2_: venous partial carbon dioxide pressure
SID: strong ion difference
SIDm: measured strong ion difference
SIG: strong ion gap
*s*SID: simplified strong ion difference approach
TP: total protein
TS: total solids
USI: unmeasured strong ions

## 1 INTRODUCTION

Currently, there are 3 approaches for clinicians to investigate the acid‐base alterations of sick patients: the Henderson‐Hasselbalch equation, the Base Excess approach, and the simplified strong ion difference (*s*SID) model.^1^, ^2^ According to the *s*SID model, the plasma pH is influenced by 3 independent factors: the p_a_CO_2_, the strong ion difference (SID), and the total weak acid concentration (A_tot_^−^).^3^ In the last decade, the development of point‐of‐care analyzers has permitted frequent stall‐side monitoring of a horse's blood gas and electrolyte concentrations. From these results, veterinarians often calculate the *s*SID variables to diagnose acid‐base disorders, guide medical decision‐making, and provide prognosis of sick horses. There are discrepancies in the measured concentrations of strong electrolytes, especially Na^+^ and Cl^−^, when comparing point‐of‐care technology and central laboratory analyzers in human^4^, ^5^, ^6^, ^7^ canine,^8^ and equine tertiary institutions.^9^, ^10^ Application of the *s*SID approach depends on the determination of several strong electrolytes, and inconsistencies in the measurement of individual ions can lead to imprecisions in the calculated values.^1^, ^3^ Thus, errors in interpretation and diagnosis of acid‐base imbalances diminishes the clinical utility of the *s*SID model.^11^, ^12^ Based on the published literature and our experience with several clinical cases where different analyzers were used to measure electrolytes in the same horse and the conclusion regarding acid‐base disorders using the *s*SID were inconsistent, we hypothesized that the calculated *s*SID differs considerably depending on the analyzer used to measure the strong ions and plasma proteins. The first objective of this study was to determine the level of agreement between 2 different analyzers for determining concentrations of strong electrolytes (Na^+^, K^+^, and Cl^−^) in sick horses. The second objective was to determine the level of agreement between total solids (TS) values obtained by refractometry and a colorimetric assay for determining total plasma protein (TP) concentrations in sick horses. The third and main objective of this study was to determine the impact of measuring electrolytes and plasma protein using different methodologies on the *s*SID calculations and, therefore, the diagnosis of acid‐base disorders in sick horses.

## 2 MATERIALS AND METHODS

### 2.1 Study population, case inclusion, and records review

Medical records from all horses admitted to the Large Animal Hospital of the University of Florida, College of Veterinary Medicine, from January 2018 through September of 2019, were reviewed. Horses were included if: 1) their history and physical exam indicated they were admitted because of compromised health status, 2) venous blood gas (VBG), electrolytes, TS, and a complete plasma biochemical profile (PBP) were measured upon admission using a whole blood gas analyzer (WBGA) and plasma biochemistry multianalyzer (PBMA), respectively, and 3) printed reports of the VBG and PBP results were available with date and time of the analysis.

### 2.2 Sample collection and measurement techniques

Samples for both WBGA and PBMA were collected simultaneously by venipuncture of the jugular vein. The blood was collected into plastic collection tubes containing lithium‐heparin additive. The PBMA and WBGA electrolyte concentrations were measured using a Beckman Coulter AU480 Chemistry analyzer (ion‐selective electrode technology based on indirect potentiometry) and Nova Biomedical pHO Ultra Stat Profile (ion‐selective electrode technology based on direct potentiometry), respectively. The TP was measured with a colorimetric assay based on a modification of the Weichselbaum method when using the PBMA. The WBGA TS were determined using refractometry.

### 2.3 Data collection

Institution electronic medical record systems were reviewed for data on horse signalment, presenting complaint, final diagnosis, outcome. The following data were extracted from the VBGA and electrolyte analyses: pH, P_v_CO_2_ (mm Hg), HCO_3_^−^ (mmol/L), blood [Na^+^], [K^+^], [Cl^−^], and [l‐lactate^−^] (all in mmol/L) and TS (g/dL). Data extracted from the PBMA included plasma [Na^+^] (mmol/L), [K^+^] (mmol/L), [Cl^−^] (mmol/L), and TP (g/dL).

### 2.4 Calculations

The *s*SID variables were calculated as strong ion difference using 4 electrolytes as (SID_4_) = (Na^+^ + K^+^) − (Cl^−^ + l‐lactate^−^); the total nonvolatile buffer ion concentration in plasma (A_tot_) = [0.22] × TP (g/dL); and the unmeasured strong ions (USI) = SID − HCO_3_^−^ − A_tot_/(1 + 10^[6.65‐pH]^).^13^ The SID_4_ and USI concentrations were calculated using the measured values of [Na^+^], [K^+^], and [Cl^−^] obtained from each analyzer. The values of pH, HCO_3_^−^and l‐lactate^−^ used in all calculations were obtained from the WBGA. The A_tot_ was calculated using the TS and TP obtained from WBGA and PBMA measurements.

### 2.5 Definitions

The acid‐base disorders were defined when the following variables were outside of the following reference ranges: SID_4_ (38 to 46 mmol/L), A_tot_ (12 to 16 mmol/L), and USI (−2 to 2 mmol/L). The SID_4_ acidosis was defined as a SID_4_ < 38 while alkalosis was a SID_4_ > 46 mmol/L; Hyperproteinemic acidosis was defined as A_tot_ > 16 mmol/L and hypoproteinemic alkalosis when A_tot_ < 12 mmol/L; USI alkalosis was defined as USI < −2 mmol/L while USI acidosis when USI > 2 mmol/L.^13^, ^14^ Similarly, the electrolyte and plasma protein abnormalities were determined when the values were outside of the reference ranges published elsewhere.^15^

### 2.6 Statistical analysis

All statistical analyses were completed using available software (MedCalc Statistical Software version 16.4.3 (MedCalc Software bv, Ostend, Belgium; https://www.medcalc.org; 2016, R R Core Team [2017]. R: A language and environment for statistical computing. R Foundation for Statistical Computing, Vienna, Austria. URL https://www.R-project.org/). Descriptive statistics were generated for all variables in the data set. Normally distributed data are presented as mean and SD. Nonnormally distributed data are presented as median and interquartile range. Normality of the data was tested by the Shapiro‐Wilk test. The Wilcoxon Signed Rank Test was used to compare values measured and calculated using each of the 2 analyzers. The Spearman rho correlation was also calculated as a robust index of correlation between both analyzers because the assessed data was nonnormally distributed.

The agreement and reliability between the 2 analyzers were assessed using different but complementary approaches. Nonparametric Passing‐Bablok regression and Bland‐Altman analysis^16^ were performed to determine the level of agreement between the 2 analyzers for measurement of Na^+^ + K^+^, Cl^−^, and calculation of SID_4_ and USI. For the Passing‐Bablok regression, the intercept (*I*) establishes a systematic difference between the 2 analyzers when the 95% CI does not include 0. The slope (*S*) reflects proportional bias when the 95% CI does not include 1. Differences were plotted using Bland‐Altman analysis, the difference between the 2 analyzers was plotted on the y‐axis against the mean of the WBGA and PBMA value on the x‐axis. The limits of agreement (upper and lower) were calculated from the bias 1.969 × SD.

Weighted Kappa coefficient test was used to evaluate the level of agreement between the 2 analyzers in detecting acid‐base disorders using prespecified cut‐offs. The Kappa agreement was judged as poor when 0 ≤ κ ≤ 0.40, fair when 0.41 ≤ κ ≤ 0.59, good when 0.60 ≤ κ ≤ 0.74, and excellent 0.75 ≤ κ ≤ 1.^17^ The McNemar test was used to determine if the proportion of detected electrolyte and acid‐base disorders was the same between the 2 analyzers. Significance was defined as a *P*‐value <.05.

Intraclass correlation coefficient (ICC) was also used to determine the reliability between the SID_4_ and USI calculations from the 2 analyzers and was interpreted as: ICC ≤ .5 = poor indicator of reliability; .5 < ICC ≤ .75 = moderate reliability; .75 < ICC ≤ .9 = good reliability; and >.9 = excellent reliability.^18^

## 3 RESULTS

### 3.1 Horses

A total 124 horses met the inclusion criteria, 75 (60%) males and 49 (40%) females. The median age was 10 years (range 1 day to 30 years). Horses were from 17 different breeds with American Quarter Horse (n = 39, 31%) and Thoroughbreds (n = 22, 18%) being the most represented. Most of the horses were presented for evaluation of gastrointestinal (n = 65, 52%) and respiratory (n = 19, 15%) diseases. At admission, 45/124 (36%) horses had fever, 77 (62%) tachycardia, and 59 (48%) tachypnea. The complete blood cell count results were available for 122 horses wherein 44 (36%) had an abnormal total white blood cell count (either leukocytosis or leukopenia). A total of 83 (67%) horses were discharged from the hospital, 36 (30%) were euthanized, and 5 (3%) died during hospitalization.

### 3.2 Sodium, potassium, chloride measurements

**FIGURE 1:**
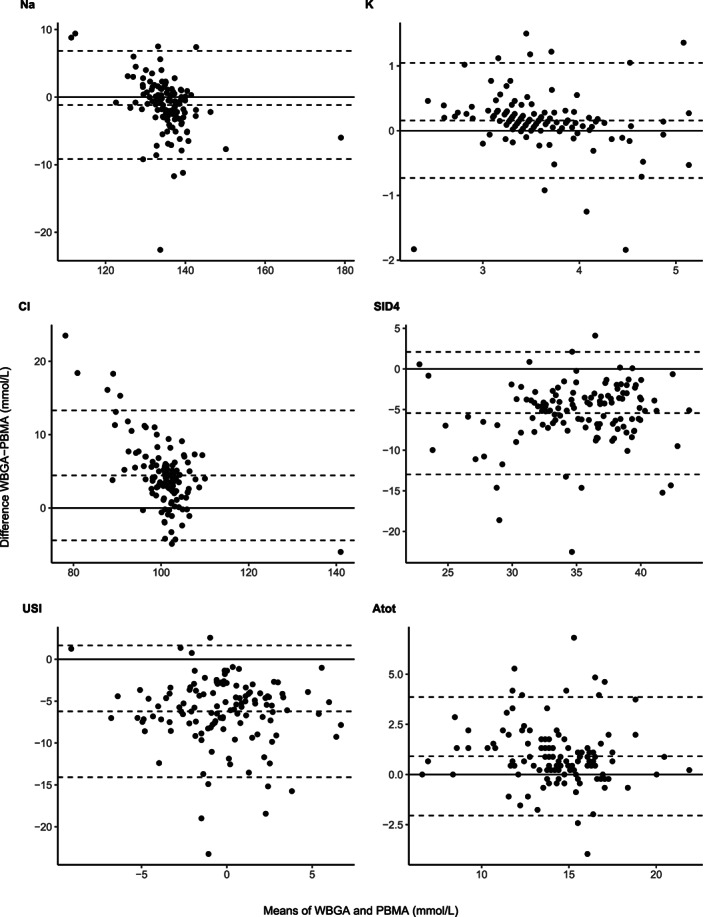
Bland‐Altman agreement plot between whole blood gas analyzers (WBGA) and plasma biochemistry multianalyzer (PBMA) for measurement of Na^+^, K^+^ and Cl^−^ and calculation of the strong ion difference (SID_4_) and unmeasured strong ions (USI)

Data for Na^+^, K^+^, and Cl^−^ were available for 124 horses. The median (25%‐75% IQR) values for Na^+^, K^+^, Cl^−^, SID_4_, and USI are presented in Table 1. The measurements of Na^+^, K^+^, Cl^−^, SID_4_, and USI were significantly different between the 2 analyzers (*P* < .01) (Table 1). The proportion of horses detected with electrolyte abnormalities is presented in Table 2. Of interest, PBMA detected more horses with hypochloremia (n = 103, 83%) than WBGA (n = 74, 60%), but WBGA detected more horses with hyperchloremia (n = 6, 5%) than PBMA (n = 1, 1%) (*P* < .001). There were no differences in the proportion of horses detected with Na^+^ or K^+^ abnormalities with each analyzer. The results of Passing‐Bablok regression indicated significant methodologic differences and proportional error in measuring Na^+^, K^+^, and Cl^−^. The Bland‐Altman plot indicated that the mean bias (95% limits of agreement) for Na^+^, Cl^−^, between the 2 analyzers were −1.2 mmol/L (−9.2 to 6.8) and 4.4 mmol/L (−4.4 to 13), respectively (Table 3 and Figure 1).

**TABLE 1:** Median (25%‐75% IQR) values for whole blood and plasma electrolytes, total solids (TS), and total plasma protein (TP) and calculated simplified strong ion difference obtained from 124 sick horses using both a whole blood gas analyzers (WBGA) and plasma biochemistry multianalyzer (PBMA)

	WBGA	PBMA	
Variable	Median (25%‐75%)	Median (25%‐75%)	*P*‐value
Na^+^ (mmol/L)	134 (132 to 137)	136 (133 to 139)	.008
K^+^ (mmol/L)	3.6 (3.4 to 4)	3.4 (3.1 to 3.8)	.003
Cl^−^ (mmol/L)	103 (100 to 104)	99 (96 to 100)	<.001
TS‐TP^a^ (g/dL)	6.7 (6.2 to 7.5)	6.4 (5.7 to 7.2)	.01
SID_4_ (mmol/L)	33 (30 to 36)	38 (35 to 41)	<.001
USI (mmol/L)	−2.9 (−5.8 to −1)	3 (0.6 to 5)	<.001
A_tot_ (mmol/L)	14 (13 to 16)	14 (13 to 16)	.009

*Note*: P‐values obtained using the Wilcoxon signed rank test.

Abbreviations: A_tot_ the total nonvolatile buffer ion concentration in plasma; SID_4_, strong ion difference; TP, total plasma protein; TS, total solids; USI, unmeasured strong ions.

^a^ TS and TP were measured using a refractometer and the PBMA, respectively.

**TABLE 2:** Prevalence of electrolyte and total protein abnormalities of 124 sick horses detected using the simplified strong ion difference approach after determining electrolytes using a whole blood gas analyzers (WBGA) and plasma biochemistry multianalyzer (PBMA)

	Na^+^		K^+^		Cl^−^		TS‐TP^a^	
	WBGA	PBMA	WBGA	PBMA	WBGA	PBMA	Refract	PBMA
Normal	99 (80%)	96 (78%)	117 (94%)	115 (93%)	103 (83%)	74 (60%)	98 (79%)	95 (77%)
Low	24 (19%)	25 (20%)	1 (1%)	2 (2%)	25 (12%)	49 (39%)	12 (10%)	23 (18%)
High	1 (1)	3 (2%)	6 (5%)	7 (5%)	6 (5%)	1 (1%)	14 (11%)	6 (5%)
*P*‐value	.56		.91		<.001		.002	

*Note*: P‐values obtained from the McNemar's test. Reference ranges (15): Na^+^ = 132‐146 mmol/L; K^+^ = 2.4‐4.7 mmol/L; Cl^−^ = 99‐109 mmol/L; TP = 5.2‐7.9 g/dL.

Abbreviations: Cl^−^, chloride; K^+^, potassium; Na^+^, sodium; Refract, refractometry; TP, total plasma protein; TS, total solids.

^a^ TS and TP were measured using a refractometer and the PBMA, respectively.

**TABLE 3:** Spearman Rho correlation coefficient, Passing‐Bablok regression (intercept, slope), and Bland‐Altman plots (bias, 95% limits of agreement) values for whole blood and plasma electrolytes, total solids, total plasma protein, and calculated simplified strong ion difference obtained from 124 sick horses using both a whole blood gas analyzers (WBGA) and plasma biochemistry multianalyzer (PBMA)

Factor	Spearman rho (*P*‐value)	Intercept (95% CI)	Slope (95% CI)	Bias (95% limits of agreement)
WBGA vs PBMA				
Na^+^ (mmol/L)	0.677 (<.001)	37.2 (23 to 47)	0.72 (0.64 to 0.82)	−1.2 (−9.2 to 6.8)
K^+^ (mmol/L)	0.736 (<.001)	0.80 (0.55 to 1.02)	0.81 (0.75 to 0.88)	0.16 (−0.73 to 1.05)
Cl^−^ (mmol/L)	0.677 (<.001)	37.9 (27.2 to 48.6)	0.66 (0.55 to 0.76)	4.4 (−4.4 to 13)
TS‐TP (g/dL)	0.813 (<.001)	0.90 (0.30 to 1.41)	0.91 (0.83 to 1)	0.4 (−0.9 to 1.8)
SID_4_ (mmol/L)	0.671 (<.001)	−8.242 (−15 to −2.10)	1.088 (0.92 to 1.27)	−5.4 (−13 to 2)
USI (mmol/L)	0.348 (<.001)	−5.56 (−6.32 to −5.09)	0.966 (076 to 1.20)	– 6.2 (−14 to 1.7)
A_tot_ (mmol/L)	0.813 (<.001)	1.980 (0.66 to 3.11)	0.916 (0.83 to 1)	0.9 (−2 to 3.9)

Abbreviations: A_tot_, the total nonvolatile buffer ion concentration in plasma; SID_4_, strong ion difference; TP, total plasma protein; TS, total solids; USI, unmeasured strong ions.

### 3.3 Total solids and total protein measurements

**FIGURE 2:**
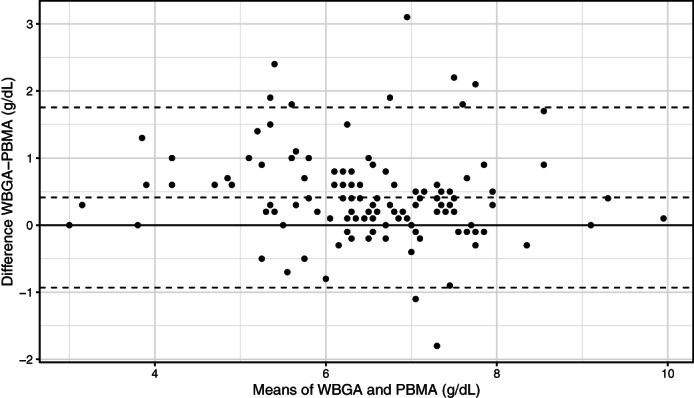
Bland‐Altman agreement plot between refractometry and plasma biochemistry multianalyzer (PBMA) for measurement of total solids and total plasma protein, respectively

The median (25%‐75% IQR) values for TS and TP are presented in Table 1. The proportion of horses detected with TS and TP abnormalities is presented in Table 2. The measurement of TS and TP was significantly different between the refractometer and plasma biochemistry analysis (*P* < .01) (Table 1). Methodologic differences, but not proportional error in measuring TS and TP using the refractometer or chemical analyzer, were identified (Table 3 and Figure 2).

### 3.4 *s*SID calculation and acid‐base disorder diagnosis

Data for SID_4_, USI, and A_tot_ were available for 124 horses. The median (25%‐75% IQR) values for *s*SID variables are presented in Table 1. The measurements of SID_4_ and USI were significantly different between the 2 analyzers (*P* < .001) (Table 1). The measurement of A_tot_ was also significantly different between the 2 different techniques (*P* = .009) The proportion of horses diagnosed with SID_4_, USI, and A_tot_ acid‐base disorders using the measured electrolytes from each machine is displayed in Table 4. The proportion of horses diagnosed with SID_4_ acidosis was significantly greater when using the values of Na^+^, K^+^, and Cl^−^ from the WBGA (85%) analyzer compared to the PBMA (44%). The proportion of horses diagnosed with USI acidosis was greater when using the values of strong electrolytes obtained from the PBMA (47%) than from the WBGA (2%) analyzer. The number of horses in which electroneutrality appeared to be violated (USI alkalosis) was higher when USI was calculated using the Na^+^, K^+^, and Cl^−^ values from WBGA (50%) than the PBMA (2%) analyzer (Table 4). The proportion of horses diagnosed with A_tot_ acidosis was greater when using the values of TP obtained from the PBMA (89%) than from the TS (63%) obtained with refractometer.

**TABLE 4:** Prevalence of acid‐base disorders of 124 sick horses detected using the simplified strong ion difference approach after determining electrolytes using a whole blood gas analyzers (WBGA) and plasma biochemistry multianalyzer (PBMA)

	SID_4_		USI^a^		A_tot_	
	WBGA	PBMA	WBGA	PBMA	WBGA	PBMA
Normal	18 (15%)	68 (55%)	60 (48%)	64 (51%)	46 (37%)	14 (11%)
Acidosis	106 (85%)	54 (44%)	2 (2%)	58 (47%)	78 (63%)	110 (89%)
Alkalosis	0 (N/A)	2 (1%)	62 (50%)	2 (2%)	0 (N/A)	0 (N/A)
*P*‐value	<.001		<.001		<.001	

*Note*: P‐values obtained from the McNemar's test analysis.

Abbreviations: A_tot_, the total nonvolatile buffer ion concentration in plasma; SID_4_, strong ion difference; USI, unmeasured strong ions.

^a^ USI alkalosis indicates that the electroneutrality law appeared to be violated.

**FIGURE 3:**
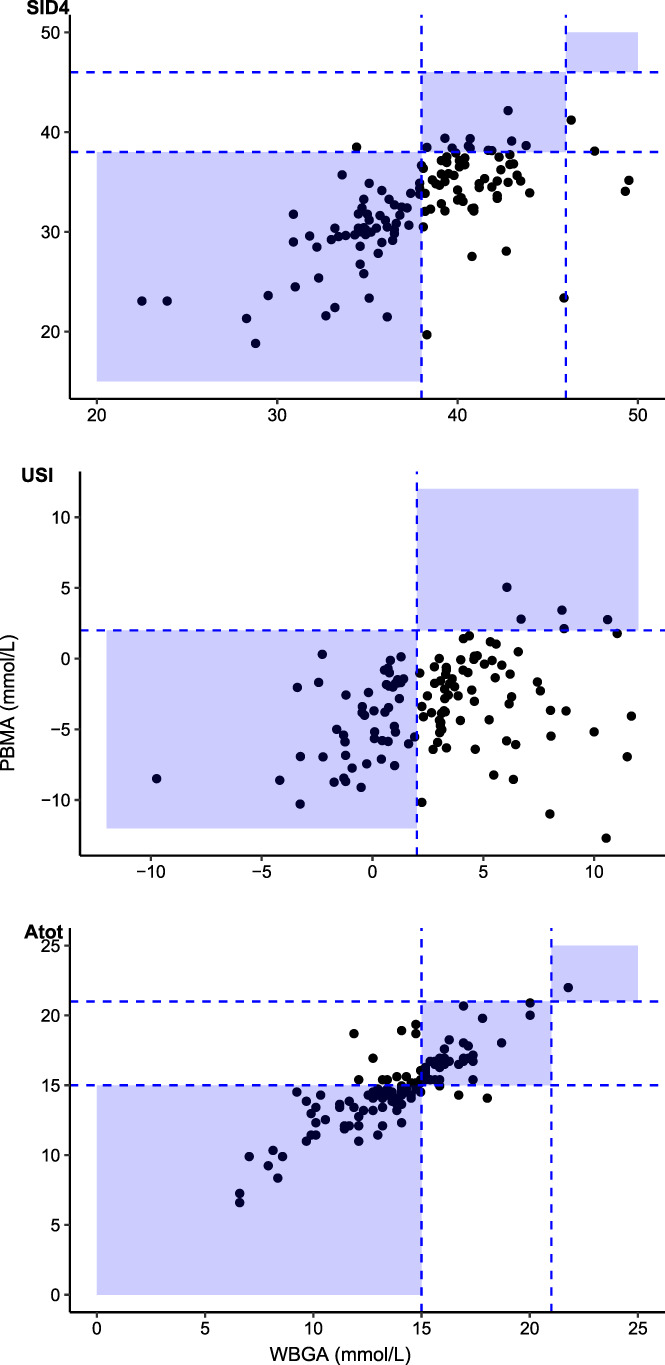
Clinical interpretation of agreement between whole blood gas analyzers (WBGA) and plasma biochemistry multianalyzer (PBMA) when assessing strong ion difference (SID_4_), unmeasured strong ions (USI); and the total nonvolatile buffer ion concentration in plasma (A_tot_) measured as total solids or totals plasma proteins. The blue shaded area indicates the area of agreement between the 2 analyzers using established clinical thresholds

The Passing‐Bablok regression showed significant methodologic differences, but not proportional error, in the calculated values of SID_4_, USI, and A_tot_ (Table 3 and Figures 2 and 3). The Bland‐Altman plot indicated that the mean bias (95% limits of agreement) for SID_4_, USI, and A_tot_ between the 2 analyzers or techniques were: −5.4 mmol/L (−13 to 2), −6.2 mmol/L (−14 to 1.7), and 0.9 mmol/L (−2 to 3.9), respectively (Table 3 and Figure 1).

Kappa coefficient analysis showed poor agreement between WBGA and PBMA for detection of SID_4_ (κ = 0.20, 95%CI, 0.1 to 0.31) or USI abnormalities (κ = −0.04, 95%CI, −0.11 to 0.02). Kappa coefficient analysis also revealed a poor agreement between refractometry and chemistry analysis for detection of A_tot_ abnormalities (κ = 0.33, 95%CI, 0.18 to 0.48). The ICC revealed poor reliability of .55 (95%CI: −0.2 to 0.8), .2 (95%CI: −0.15 to 0.48), and .88 (95%CI: 0.89 to 0.941) for the measurement of the SID_4_, USI, and A_tot_, respectively, using the different analyses.

## 4 DISCUSSION

This study revealed significant differences in measuring concentrations of Na^+^ and Cl^−^ using a whole blood gas and electrolyte analyzer compared with a plasma biochemistry multianalyzer. These differences affected the calculation of SID_4_ and USI. Similarly, determination of the TS and TP using 2 different methodologies affected the calculation of the A_tot_. The impact of the different methodologies on the *s*SID variables resulted in poor overall agreement between the analyzers and techniques to diagnose acid‐base disorders in sick horses.

The reasons for the differences in the measured electrolyte concentrations between analyzers are discussed elsewhere.^11^, ^19^, ^20^ Briefly, time between sample collection and processing by each analyzer is often different. Samples analyzed with the WBGA are generally processed within minutes after blood withdrawal, while samples analyzed with PBMA require additional time to be processed which could explain the differences in K^+^, but not in Na^+^ or Cl^−^ concentrations.^21^ The type of preferred sample (whole blood vs plasma) used in each analyzer could have also affected the concentration of strong ions in the samples. However, a previous study measured plasma electrolyte concentration using a point‐of‐care blood gas analyzer and a central laboratory chemistry analyzer and lower plasma Na^+^ and higher Cl^−^ concentrations were measured with the point‐of‐care analyzer,^22^ as was the case when determining strong ions concentrations in whole blood in this study. This suggests that, at least in part, the differences in electrode activity can impact the electrolyte concentration variability.^10^, ^19^, ^20^ This finding is expected as the methodology differences are commonly encountered between blood gas analyzers and diagnostic laboratory equipment.

The variability in the SID_4_ and USI results between 2 analyzers can be explained by the accumulation of errors in each electrolyte measurement.^10^, ^11^, ^12^ The variability in the A_tot_ is explained by the differences in the measurement of TS and TP using either the refractometer or the chemistry analyzer. In our study, the mean differences for Na^+^ was −1.2 and 4.4 mmol/L for Cl^−^. Although these differences appeared to be small, the 95% limits of agreement of these differences extended from −9.2 to +6.8 and from −4.4 to +13 mmol/L, respectively. In human medicine, there are wide limits of agreement for the SID_m_ (−3.4 to +9.5 mmol/L and −5 to +4.7) when electrolytes are measured using point‐of‐care blood gas analyzer and was compared with central laboratory biochemistry multianalyzer.^11^, ^12^ In horses, there is a large limit of agreement (−3.6 to +11.5) for SID_3_ (measured as SID_3_ = Na^+^ + K^+^ − Cl^−^) when the electrolyte concentrations were determined using a blood gas analyzer and automated multianalyzer system.^10^ These broad limits of agreement have a compounding effect on the calculation of SID_4_ (ie, 95% limits of agreement of −13 to +2 in this study) and, therefore, in the USI, as changes in SID_m_ will produce a change of similar magnitude in the USI.^2^, ^3^, ^11^, ^12^ In addition, the limits of agreement of A_tot_ also exert effect in the calculation of the USI. There is a wide range of USI (measured as SIG) in healthy foals,^22^ likely due to inaccuracies in the measurement of strong ions. This wide range is markedly different to the commonly reported normal value in horses, cattle and humans of USI of −2 to +2 mmol/L.^13^ Results from this study, and those from previous investigations, highlighted the difficulties in obtaining both “normal” values for SID_m_ and USI and, accurate estimations of SID_m_ and USI in sick horses. These difficulties are due to variation in electrolytes, TS and TP concentration among individuals, and laboratory variation in measurements within any horse.

From the clinician's perspective, the differences in the measurement of strong ions and calculation of SID_4_ and USI are not informative, and more importantly, can be clinically misleading. This study showed that variability in the *s*SID calculations can affect either the diagnosis of acid‐base disorders of sick horses or the evaluation of their disease state.^23^ For example, using WBGA, 85% of the horses were diagnosed with a SID_4_ acidosis and 15% had a normal SID_4_, whereas using PBMA, 44% and 55% of the horses were diagnosed with a SID_4_ acidosis and normal SID_4_, respectively. These differences in the diagnosis of acid‐base disorders could have diagnostic and therapeutic implications when a disorder is detected by 1 analyzer but not the other. For instance, if a horse has SID_4_ acidosis, likely due to hyperchloremia, renal function assessment would be indicated,^24^, ^25^ loop diuretics could be administered,^26^ or chloride‐rich fluids might be avoided.^27^, ^28^, ^29^ Similar observations should be considered when evaluating horses with SID_4_ alkalosis. Clinicians using the *s*SID methodology to determine acid–base balance in sick horses should be cognizant that the decision‐making process regarding the horse's diagnostic and treatment approach can largely be affected by the analyzer used to measure strong ion concentrations.

The variability in the calculations of the horse's USI can also have clinical implications. Diagnostic assessment could be affected in a horse with USI that were higher than initially suspected, as was the condition of almost 50% of horses. For example, an increase in USI could prompt the clinicians to further investigate the source of those USI, such as determining phosphates, citrate, d‐lactate, Krebs cycle intermediates, uremic acids and ketone bodies,^1^, ^3^, ^23^, ^30^ or assessing hepatic^31^, ^32^ and renal function.^31^, ^33^ Similarly, the USI variability between analyzers can also have prognostic implications. In human and bovine medicine, there is data suggesting that the high USI values are associated with the presence of endotoxin in diarrheic calves^34^ and that USI concentrations can predict the outcome (hospital survival) in critically ill children^35^ and calves with diarrhea.^30^ In horses, the concentration of USI (measured as strong ion gap, SIG) was greater in nonsurviving than surviving hospitalized foals.^22^ This is of importance as differences in the USI values can lead clinicians to make different conclusions about the diagnosis and prognosis of the patient depending on the analyzer or techniques used for measurement of electrolytes and plasma proteins. Findings from this study suggest that clinicians should be aware of the methods and assays used by their laboratories to ensure that they are similar to those used in studies from which clinical guidelines and recommendations are provided.^11^, ^12^

This study has several limitations. The most notable is its retrospective design that prevents the determination of preanalytic effects including the exact time from sample collection to processing, sample quality and the collected blood volume or the volume of plasma used. All these variables could have an impact on the differences in electrolyte concentrations detected in this study. Additionally, this study only investigated the SID_4_ and USI in a large sample of sick horses from a single teaching hospital. Therefore, our results cannot be extrapolated to a different population. However, the cases admitted to our teaching hospital include a variety of sick horses similar to those admitted to other tertiary referral veterinary hospitals. This view is supported by the wide distribution of the values of the *s*SID component variables reported here. An additional limitation was that this study only compared 2 analyzers for measuring electrolytes and 2 techniques for determination of plasma proteins, limiting our conclusions to these technologies.

## CONFLICT OF INTEREST DECLARATION

Sébastien Buczinski serves as Consulting Editor for Experimental Design and Statistics for the Journal of Veterinary Internal Medicine. He was not involved in review of this manuscript.

## OFF‐LABEL ANTIMICROBIAL DECLARATION

Authors declare no off‐label use of antimicrobials.

## INSTITUTIONAL ANIMAL CARE AND USE COMMITTEE (IACUC) OR OTHER APPROVAL DECLARATION

Authors declare no IACUC or other approval was needed.

## HUMAN ETHICS APPROVAL DECLARATION

Authors declare human ethics approval was not needed for this study.
